# Virtual Objective Structured Clinical Examination (OSCE) Training in the Pandemic Era: Feasibility, Satisfaction, and the Road Ahead

**DOI:** 10.7759/cureus.61564

**Published:** 2024-06-03

**Authors:** Elshazaly Saeed, Muddathir H Hamad, Abdullah N Alhuzaimi, Fadi Aljamaan, Hossameldin Elsenterisi, Heba Assiri, Khalid Alhasan, Fahad A Bashiri, Mohammad Kambal, Mahmoud Salah Khalil, Hamza Mohammad Abdulghani, Jaffar A Al-Tawfiq, Ayman Al-Eyadhy, Mohamad-Hani Temsah

**Affiliations:** 1 Department of Pediatrics, Prince Abdullah Ben Khaled Coeliac Disease Research Chair, College of Medicine, King Saud University, Riyadh, SAU; 2 Department of Pediatric Neurology, King Saud University Medical City, Riyadh, SAU; 3 Department of Cardiac Science, College of Medicine, King Saud University, Riyadh, SAU; 4 Department of Pediatric Cardiology, King Faisal Specialist Hospital and Research Centre, Riyadh, SAU; 5 Department of Critical Care Medicine, College of Medicine, King Saud University, Riyadh, SAU; 6 Department of Medicine, Al Rayan Hospital, Dr. Sulaiman Al Habib Medical Group, Riyadh, SAU; 7 Department of Pediatrics, King Saud University Medical City, Riyadh, SAU; 8 Department of Pediatrics, Dr. Sulaiman Al Habib Medical Group, Riyadh, SAU; 9 Department of Kidney and Pancreas Health Center, King Faisal Specialist Hospital and Research Centre, Riyadh, SAU; 10 Department of Pediatrics, College of Medicine, King Saud University, Riyadh, SAU; 11 Department of Pediatrics, Specialized Medical Center Hospital, Riyadh, SAU; 12 Department of Medical Education, College of Medicine, King Saud University, Riyadh, SAU; 13 Department of Specialty Internal Medicine and Quality, Johns Hopkins Aramco Healthcare, Dhahran, SAU; 14 Department of Medicine, Indiana University School of Medicine, Indianapolis, USA; 15 Department of Medicine, Johns Hopkins University School of Medicine, Baltimore, USA; 16 Department of Pediatrics, Pediatric Intensive Care Unit, King Saud University Medical City, Riyadh, SAU; 17 Department of Family and Community Medicine, Evidence-Based Research Chair, College of Medicine, King Saud University, Riyadh, SAU

**Keywords:** skills and simulation training, virtual training, zoom platform, pediatrics education, remote medical training during lockdown, digital transformation in medical education, objective structured clinical examination training, medical education during infectious disease outbreak, covid-19 pandemic, virtual osce training

## Abstract

Introduction: Objective Structured Clinical Examinations (OSCEs) are essential assessments for evaluating the clinical competencies of medical students. The COVID-19 pandemic caused a significant disruption in medical education, prompting institutions to adopt virtual formats for academic activities. This study analyzes the feasibility, satisfaction, and experiences of pediatric board candidates and faculty during virtual or electronic OSCE (e-OSCE) training sessions using Zoom video communication (Zoom Video Communications, Inc., San Jose, USA).

Methods: This is a post-event survey assessing the perceptions of faculty and candidates and the perceived advantages and obstacles of e-OSCE.

Results: A total of 142 participants were invited to complete a post-event survey, and 105 (73.9%) completed the survey. There was equal gender representation. More than half of the participants were examiners. The overall satisfaction with the virtual e-OSCE was high, with a mean score of 4.7±0.67 out of 5. Most participants were likely to recommend e-OSCE to a friend or colleague (mean score 8.84±1.51/10). More faculty (66.1%) than candidates (40.8%) preferred e-OSCE (P=0.006).

Conclusion: Transitioning to virtual OSCE training during the pandemic proved feasible, with high satisfaction rates. Further research on virtual training for OSCE in medical education is recommended to optimize its implementation and outcomes.

## Introduction

Objective Structured Clinical Examinations (OSCEs) are performance-based assessments used to evaluate clinical skills through a series of stations with standardized patients or mannequins to measure students’ clinical skills to assess their competencies [1]. Since the beginning of the COVID-19 pandemic, medical education activities have been impacted globally, and many institutes have implemented virtual formats or distance learning for academic activities [2-5]. Transmission of SARS-CoV-2 was decreased by social distancing strategies, but that resulted in an urgent need to adopt new methods of delivering and assessing medical education [3]. Electronic or virtual OSCE (e-OSCE) can provide a valuable alternative to face-to-face OSCE and has been widely accepted among medical students and examiners [6].

There are disadvantages to virtual clinical examinations (Integrated Structured Clinical Examinations (ISCEs) or OSCEs) compared to in-person examinations. These disadvantages include challenges in assessing skills including physical examinations or procedural skills, and disciplinary concerns like dishonesty among students when conducting assessments off-campus [7]. Despite these challenges, balancing the changing needs and technological advancements of undergraduate medical curricula during the pandemic remains vital. During the COVID-19 pandemic, online ISCEs/OSCEs provided an opportunity to supplement other forms of summative clinical competency assessments, and their benefits may extend beyond the pandemic [8].

The pediatric OSCE training review course aims to prepare board-eligible pediatric residents for the final clinical exam and improve their performance. It was designed to consolidate the knowledge of the candidates in history taking, physical examination, data interpretation, evidence-based medicine, communication skills and management plans for most of the common childhood disorders. This review course has been running annually since 2008 at the College of Medicine, King Saud University [9]. In 2020, the course organizers decided to run the course virtually, as e-OSCE, through the Zoom video-conferencing platform (Zoom Video Communications, Inc., San Jose, USA) because of the COVID-19 pandemic. With careful planning, e-OSCEs were successfully administered. The Zoom platform was chosen due to familiarity among examiners and students as well as the utilization of the “breakout-out room” function, which facilitates private mini-sessions [1]. This research describes the process of transforming OSCE training into virtual form, assessing its feasibility and the experience of the involved candidates and faculty members.

## Materials and methods

Setting

This study was conducted in Saudi Arabia, Riyadh city at King Khaled University Hospital, which is a multi-disciplinary healthcare facility with general and subspecialty medical services that provides primary, secondary and tertiary care. The examiners were selected from national and international panels.

Objectives of the study

To describe the process of conducting the virtual clinical course using Zoom video communication. This includes identifying the advantages and disadvantages of using virtual courses and exploring the characteristics of the participants and their preferred OSCE style during the COVID-19 pandemic.

The first step, three months before the beginning of the course, the registration was opened for the candidates via an online registration form. The organizing committee members arranged the managerial and technical issues. Six virtual meetings were held to discuss the newly proposed eOSCE course content and logistics, including the development of the course structure, selection of appropriate clinical scenarios, allocation of examiners and candidates, technical requirements, and contingency planning for potential issues that might arise during the virtual sessions. Approval was obtained from the Dean's office, College of Medicine, and the Pediatric Department Chairman. Senior examiners who were previously contributing to pre-COVID-19 OSCE training workshops were invited from various localities, both nationally and internationally, to empower the training experience for the candidates. The examiners were pediatric consultants with at least two years of clinical experience.

Then, one week before the starting date, the course instructions were determined, and WhatsApp groups were created for the examiners, candidates and the organizing committee to facilitate communication. We used the WhatsApp platform because it was a commonly used communication tool in Saudi Arabia at that time. The organizing team selected the Zoom platform because of the advanced feature of break-out rooms, allowing the participants to move from one station to another without multiple signs in, as well as being one of the two-way e-learning approaches for teaching and student learning that were rolled out at our teaching institute during the early pandemic crisis [10].

One day before the course, a simulation Zoom meeting was held to test and fix any problems that might face the examiners and candidates and to discuss the regulations of the course. The response was excellent, with almost complete attendance from both examiners and candidates.

The candidates and examiners were asked to download and test the Zoom application on their laptops or PCs. All participants were asked to use their full names to join and to keep their cameras on all the time to facilitate and enhance the telecommunication experience.

Finally, the candidates were divided into two online groups: one for e-OSCE stations and the other for virtual oral/clinical sessions. Two dedicated Zoom links were created: one for the e-OSCE stations and the other for the virtual clinical and oral sessions. The breakout room function was used to create 10 rooms for the e-OSCE stations and three rooms for the oral sessions.

e-OSCE stations

The designated candidates were admitted to the virtual waiting room then each three was distributed to the assigned stations. One acted as examinee and the other two candidates as observers. The examiners were asked to share the clinical scenario on the screen at the beginning of each station. Eight minutes were allotted for the questions and the e-OSCE scenario, followed by two minutes of feedback for all three candidates. Afterward, the Zoom host moved the examiner to the next breakout room, and the next eOSCE station began. The roles rotated, with the previous examinee becoming an observer, and another candidate from the group taking the role of the examinee in the new e-OSCE cycle.

Clinical/oral sessions

The candidates were distributed into three virtual rooms. Each clinical session was held for 30 minutes, after which the examiner was rotated to the other room.

Expert examiner sessions

In order to have in-depth discussions with the board exam candidates, all of them were divided into three groups for afternoon sessions, with each session lasting for 45 minutes.

At the end of the course, an online survey was distributed to all the faculty examiners and the candidates to assess their satisfaction, experience, and obstacles.

Statistical data analysis

The mean and standard deviation were used to describe the continuous measured variables and the frequencies and percentages were applied to describe categorically measured variables. The Kolmogorov-Smirnov statistical normality test with Histograms was used to assess the statistical normality of continuous variables and the Levene's test was used to assess the equal variance statistical assumption. The multiple response dichotomies analysis was used to describe the respondents’ perceptions about the e-OSCE course measured with multiple simultaneous selections/options. The Chi-squared test of independence was used to assess the associations between categorically measured variables and the independent samples t-test was used to assess the statistical significance of mean differences of examinee and faculty members on key perceptions about the e-OSCE program across the levels of categorically measured binary variables. The Statistical Package for the Social Sciences (IBM SPSS Statistics for Windows, IBM Corp., Version 21.0, Armonk, NY) was used for statistical data analysis and the statistical significance was considered at 0.050 level.

## Results

Out of 142 participants (68 faculty and 74 candidates) in the course who were invited to fill in the survey, 105 members (overall response rate of 73.9%) completed the post-event survey. Almost both sex is represented equally, one-third of them were 30 years of age or younger. Slightly more than 50% of participants were examiners (Table 1).

**Table 1:** Respondents' socio-demographic and professional characteristics (N=105)

Variable	Category	Frequency	Percentage
Sex	Male	54	51.4
	Female	51	48.6
Age	18-30 years	36	34.3
	31-40 years	26	24.8
	41-50 years	21	20
	>=51 years	22	21
Clinical role	Faculty examiner	56	53.3
	Candidate	49	46.7

The attendee’s overall satisfaction with the e-OSCE was high with a mean score of 4.7±0.67 out of 5 scores, and the majority stated they would recommend e-OSCE for their colleagues. Almost all participants have previous experience with Zoom and about half of them have attended different online webinars. More than half of the participants indicated that they prefer attending a virtual OSCE during the COVID-19 pandemic. At the same time, 22 (21%) stated they prefer classic face-to-face courses and 26 (24.8%) had no preference between virtual and classic courses (Table 2).

**Table 2:** The virtual OSCE course attendees’ perceptions about the medical online exam-training course OSCE: Objective Structured Clinical Examinations

Items	Frequency	Percentage
Your overall satisfaction with the OSCE course, mean (SD) 1-5 satisfaction score	-	4.51 (0.67) Mean (SD)
Previous experience with teleconference		
Zoom	102	97.1
Face-time	21	20
Webinars	51	48.6
work-related online meetings	53	33.3
Online learning	43	41
Other methods	7	6.7
How likely is it that you would recommend virtual OSCE course (e-OSCE) to a friend or colleague? Mean (SD) 1-10 Likelihood rating scale	-	8.84 (1.51) Mean (SD)
In regards to your previous experience with “classic face-to-face” clinical OSCE, what is the preferred OSCE style for you during the COVID-19 pandemic?		
Virtual OSCE (e-OSCE) is preferred	57	54.3
Classic face-to-face is preferred	22	21
Both are equally preferred by me	26	24.8
How do you think these remote clinical exams (e-OSCE) affected the quality of the resident’s assessment?		
Similar assessment to the face-to-face OSCE	62	59
Better assessment than face-to-face OSCE	11	10.5
Worse assessment than face-to-face OSCE	32	30.5
How comfortable did you feel participating in this remote clinical exam (via Zoom or any other similar application)?	-	4.18 (0.82) Mean (SD)
Extremely comfortable	42	40
Very comfortable	45	42.9
Somewhat comfortable	13	12.4
Not so comfortable	5	4.8
Doing remote video assessment during COVID pandemic decreased my anxiety-mean (SD)	-	3.50 (0.91) Mean (SD)
Strongly disagree	3	2.9
Disagree	11	10.5
Neither agree nor disagree	33	31.4
Agree	48	45.7
Strongly agree	10	9.5
Video conferencing as assessment tool for the pediatric course should be incorporated in next year’s courses.	-	3.36 (1.15) Mean (SD)
Strongly disagree	8	7.6
Disagree	19	18.1
Neither agree nor disagree	19	18.1
Agree	45	42.9
Strongly agree	14	13.3
Do you suggest continuing remote resident’s assessment (via Zoom or similar platforms) after the COVID-19 crisis?		
Yes	22	21
No	83	79

Regarding the quality of the resident's assessment using e-OSCE, almost two-thirds (n=73, 69.5%) perceived it as either similar or even better than the face-to-face assessment. Of the participants, 42 (40%) felt extremely comfortable and 45 (42.9%) felt very comfortable during the e-OSCE.

Furthermore, 58 (55.2%) agreed that doing remote video assessment during the COVID-19 pandemic relieved their anxiety (mean 3.5/5 (SD) 0.91) on a scale of 1-5 likelihood rating. Two-fifths of the participants advised incorporating video conferencing as an assessment tool for the pediatric course in next year's courses. However, most participants (n=83, 79%) did not suggest continuing remote resident's assessment after the COVID-19 crisis.

Advantages and disadvantages of using virtual OSCE course

**Figure 1:**
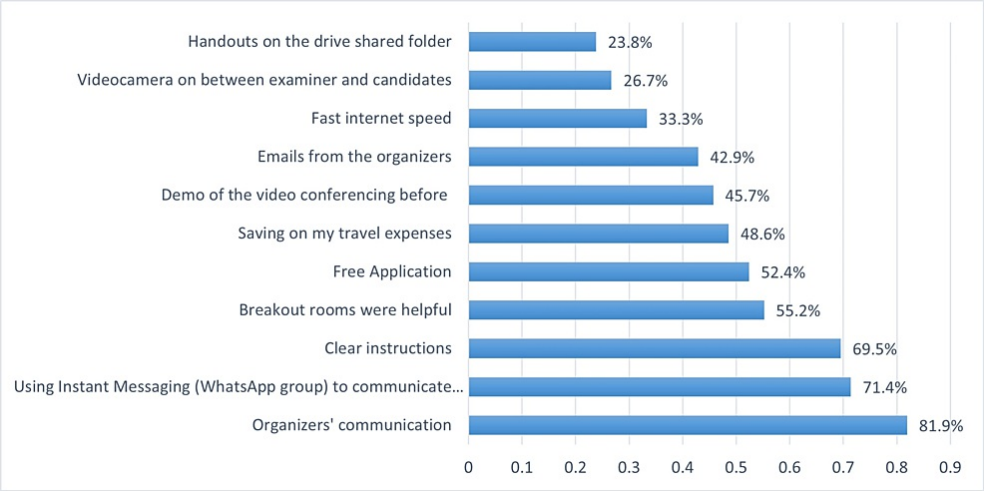
Perceived positive aspects of the virtual OSCE course OSCE: Objective Structured Clinical Examinations

**Figure 2:**
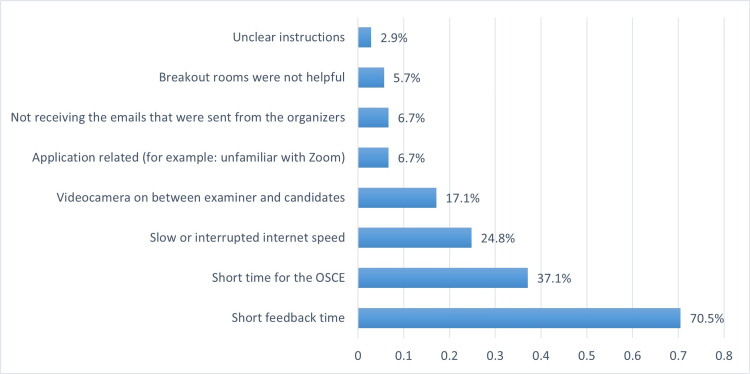
Perceived obstacles during the virtual OSCE course OSCE: Objective Structured Clinical Examinations

The positive aspects of using the virtual platform to perform OSCE were evaluated among respondents and shown in Figure 1. The organizers’ communication, using an instant messaging application (WhatsApp) group and providing clear instructions, using a free video-conferencing application and saving travel expenses were the most frequently mentioned advantages. On the other hand, the disadvantages of using the virtual OSCE are shown in Figure 2. Short time for giving the feedback, short OSCE duration and application-related obstacles were the main concerns facing the participants, in addition to unfamiliarity with break-out rooms.

Candidates' vs faculty's perceptions about the e-OSCE program/course

A bivariate comparison of examinees and faculty examiners on their perceptions of the e-OSCE program/course was performed. The majority (n=37, 66.1%) of faculty members prefer virtual OSCE. Candidates were less likely to prefer virtual OSCE (n=20, 40.8%) and more likely to think both physical and virtual courses are equally preferred (n=19, 38.8%) (P=0.006) as shown in Table 3.

**Table 3:** Bivariate comparison between examinees and faculty examiners on the perceptions about the OSCE program/course OSCE: Objective Structured Clinical Examinations

Variables	Faculty examiner n (%)	Candidate examinee n (%)	Statistics test	p-value
In regard to your previous experience with "classic face-to-face" clinical OSCE, what is the preferred OSCE style for you during the COVID-19 pandemic?			χ2(2) =10.4	0.006
Virtual OSCE (e-OSCE) is preferred	37 (66.1)	20 (40.8)		
Classic face-to-face is preferred	12 (21.4)	10 (20.4)		
Both are equally preferred by me	7 (12.5)	19 (38.8)		
How do you think these remote clinical exams (e-OSCE) affected the quality of the resident's assessment?			χ2(2) =6.50	0.039
Similar assessment to the face-to-face OSCE	37 (66.1)	25 (51)		
Better assessment than face-to-face OSCE	2 (3.6)	9 (18.4)		
Worse assessment than face-to-face OSCE	17 (30.4)	15 (30.6)		
How comfortable did you feel participating in this remote clinical exam (via Zoom or any other similar application)?			χ2(3) =7.40	0.062
Extremely comfortable	22 (39.3)	20 (40.8)		
Very comfortable	29 (51.8)	16 (32.7)		
Somewhat comfortable	4 (7.1)	9 (18.4)		
Not so comfortable	1 (1.8)	4 (8.2)		
How likely is it that you would recommend virtual OSCE course (e-OSCE) to a friend or colleague? Mean (SD) 1-10 Likelihood rating scale	9 (1.4)	8.63 (1.66)	t (91.6) =1.24	0.217
Doing remote video assessment during the COVID-19 pandemic decreased my anxiety. Mean (SD) 1-5	3.52 (0.83)	3.434 (1.01)	t (102) =0.45	0.657
Video conferencing as an assessment tool for the pediatric course should be incorporated in next year's courses. Mean (SD) 1-5	3.43 (1.17)	3.33 (1.10)	t (102) =0.425	0.672

When participants were asked if remote clinical exams (e-OSCE) affected the quality of the resident’s assessment. Most examiners (n=37, 66.1%) and candidates (n=25, 51%) thought e-OSCE gave a similar assessment as compared to face-to-face OSCE. However, regarding assessment fairness between e-OSCE and face-to-face OSCE, nine (18.4%) candidates, as opposed to two (3.6%) examiners, perceived e-OSCE gave a better assessment than face-to-face OSCE (P value=0.039), while the majority of both groups felt both ways of assessment similar and almost third of both groups felt e-OSCE worse. In contrast, both groups, examiners and candidates did not have any significant difference in their perception of comfort participating in e-OSCE or recommending the experience to their colleagues (via Zoom or any other similar application). Additionally, both groups had similar anxiety relief of incorporating virtual platforms for e-OSCE during the COVID-19 pandemic.

## Discussion

In this study, we assessed the perception of faculty and students regarding e-OSCE. The study showed that the attendees’ overall satisfaction with e-OSCE was high, and the majority stated they were likely to recommend e-OSCE to a friend or colleague. Most participants felt either extremely comfortable or very comfortable (n=87, 82.9%) during the remote clinical examination, in addition, 58 (55.2%) agreed that doing remote video assessment during the COVID-19 pandemic decreased their anxiety. These results were in agreement with others who showed the advantages of e-OSCE over the on-campus format that include a reduction in traveling time, undertaking the exam in a comfortable environment, and the potential of scaling up with smoother logistics [11,12]. More than two-thirds of both examiners and candidates were satisfied with the quality of assessment using e-OSCE, which is in agreement with others who showed that e-OSCE could be a strong substitute for standard OSCE for assessing clinical competence and has the potential to be used even beyond the pandemic and with other studies which reported that virtual OSCE sessions improve students’ confidence in history taking, communication and data interpretation skills [10,13]. Most of the participants and examiners who have also participated in in-person OSCE examinations indicated that virtual OSCE sessions were just as engaging and interactive as in-person examinations. e-OSCE has the potential to be beneficial beyond the pandemic [11].

Over 50% of those involved expressed a preference for participating in a virtual OSCE amidst the COVID-19 outbreak. Additionally, nearly 80% of our respondents proposed that certain aspects of remote resident assessment should be retained even after the pandemic has subsided. Similarly, during the pandemic, videoconferencing became an emergent solution in the medical residency application process in Saudi Arabia [14]. It was seen as a competent and hopeful substitute for face-to-face interviews in future scenarios. The main drivers of applicant satisfaction included efficient organization, the saving of expenses, and the opportunity for candidates to effectively represent themselves. There is a clear call for further studies to refine and improve this experience in the post-pandemic era.

Our results are in agreement with others who showed that online OSCE could be a useful alternative to conventional clinical assessments in times of crises where in-person contact between students, examiners, and patients would be impossible. The inability to assess students' physical examination skills remains a significant limitation [15] and one of the drawbacks of e-OSCE is the inability to assess students’ physical examination skills, which is an understandable and unavoidable drawback of online clinical assessment in general [16]. Still, adaptations could optimize the setting, such as in a study conducted during the COVID-19 pandemic that highlighted the challenges and opportunities of transitioning to remote teaching in medical programs [17]. This research compared the efficacy of a hybrid online teaching format to traditional in-person sessions for a surgical skills class. The subsequent evaluations post-class revealed no significant difference in learning outcomes between the two teaching methods, reinforcing the idea that both online and in-person formats can be effective even for surgical skills training [17].

The positive aspects of using the virtual platform in our study include clear organizers’ communication, using a free instant messaging application, clear instructions and savings on travel expenses. These findings agreed with Dost et al., who reported that additional benefits of e-OSCE over traditional in-person group teaching include reduced travel time, which could allow students to spend more time learning, while providing similar benefits to in-person teaching but in a more comfortable environment [18]. In addition, it has been reported that e-OSCE sessions allow for larger and more frequent sessions to be held without physical constraints, such as room reservations. Students and examiners' favorable responses suggested that this approach of simulated e-OSCE delivery was acceptable and feasible for both students and teachers [18]. Additionally, research using similar virtual OSCEs has demonstrated that it is practical, cost-effective, and applicable in the current global situation, with a potential role even in the post-pandemic era [19].

On the other hand, the disadvantages of using the virtual OSCE included inadequate time for giving feedback, interrupted internet speed cases and application-related obstacles. The drawback of inadequate time could be attributed to the inadequate practice of similar exams online and due to significant differences between the virtual and in-person teaching formats. Currently, it is challenging to comment on whether online teaching can fully replace clinical face-to-face teaching [18,20]. Moreover, virtual teaching poses unique challenges, including reduced student engagement compared to in-person sessions [21].

As the landscape of medical education evolves, it is vital to acknowledge and adapt the expanding roles of large language models (LLMs), like ChatGPT and Google Bard, in shaping the future of medical training [22,23]. The integration of these new technologies into the medical curriculum training and assessment could become a transformative shift, especially as we transition from the challenges posed by the pandemic. A recent investigation into the research trends of LLMs, including ChatGPT and Google Bard, compared their publication trajectories with the early research during the COVID-19 outbreak [24]. The data, sourced from the Scopus database in July 2023, revealed a staggering 1,096 articles dedicated to ChatGPT, of which roughly one-fourth pertained to medical sciences. This surge in AI-centric research emphasizes its growing prominence and the potential implications it holds for virtual OSCE and other assessment tools. Remarkably, in a virtual OSCE focusing on obstetrics and gynecology, ChatGPT outperformed human candidates, both in scores and time needed to generate answers, further underscoring its potential in medical assessments [25,26].

While the study provides insights into the feasibility and satisfaction of e-OSCE, it has inherent limitations. The reliance on self-reported data might have introduced biases. The study's focus on a single institution and the use of one platform (Zoom) might limit the generalizability of the findings. Additionally, this research did not explore the long-term efficacy and impact of e-OSCE compared to traditional methods, nor did it assess how these differences might have influenced candidates' performance in their actual OSCE. Furthermore, future research should investigate why some participants preferred face-to-face OSCEs over e-OSCEs.

As the pandemic's shadows recede, the ascendancy of LLMs in medical research literature becomes even more pronounced, warranting more efforts to further integrate AI tools with virtual online assessments, with a call for researchers, medical educators, and policymakers to harness the novel potential of AI in healthcare, ensuring that virtual OSCEs and similar training assessments are appropriately integrated in this new era.

## Conclusions

The rapid transition to virtual OSCE training during the initial pandemic crisis showcased its potential and efficacy. The high satisfaction among participants, combined with the advantages of accessibility and technological efficiency, highlighted the strengths of the virtual approach. However, to elevate the virtual OSCE experience, addressing challenges like internet stability, refining the online modality, and comprehensive digital orientation for all participants is crucial. As we move from the post-pandemic era into the digital age and AI chatbots, more research is warranted on the integration of LLMs in virtual OSCEs.
